# Poly[bis­(μ_2_-pyrimidine-κ^2^ *N*:*N*′)bis­(seleno­cyanato-κ*N*)zinc]

**DOI:** 10.1107/S1600536811023129

**Published:** 2011-06-18

**Authors:** Jan Boeckmann, Thorben Reinert, Christian Näther

**Affiliations:** a Institut für Anorganische Chemie, Christian-Albrechts-Universität Kiel, Max-Eyth Strasse 2, D-24098 Kiel, Germany

## Abstract

The asymmetric unit of the title compound, [Zn(NCSe)_2_(C_4_H_4_N_2_)_2_]_*n*_, consists of one Zn^2+^ cation located on a special position with site symmetry 2/*m*, one seleno­cyanate anion on a mirror plane and one pyrimidine ligand on a twofold rotation axis. The zinc cation is coordinated by six N atoms of four pyrimidine ligands and two N-bonded seleno­cyanate anions in mutually *trans* orientations within a slightly distorted octa­hedral coordination environment. The Zn atoms are μ-1,3-bridged *via* the pyrimidine ligands into a polymeric layer extending parallel to (100).

## Related literature

For isotypic structures with different divalent transition metals and thio­cyanate ligands, see: Bhosekar *et al.* (2010 ▶); Lloret *et al.* (1998 ▶, 1999 ▶); Wriedt *et al.* (2009 ▶); Wriedt & Näther (2010 ▶).
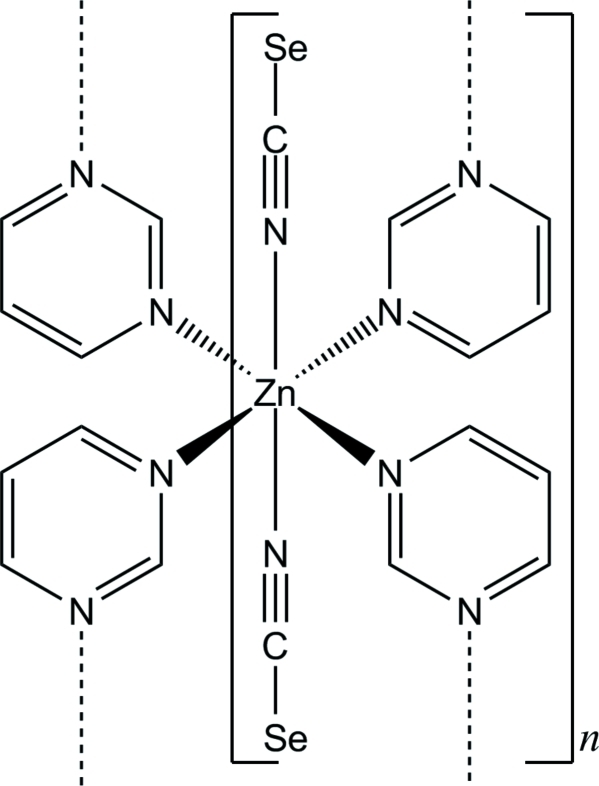


## Experimental

### 

#### Crystal data

[Zn(NCSe)_2_(C_4_H_4_N_2_)_2_]
*M* *_r_* = 435.51
Orthorhombic, 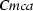

*a* = 9.4025 (9) Å
*b* = 16.7146 (10) Å
*c* = 8.7886 (5) Å
*V* = 1381.21 (17) Å^3^
*Z* = 4
Mo *K*α radiation
μ = 7.04 mm^−1^
*T* = 200 K
0.28 × 0.22 × 0.16 mm

#### Data collection

Stoe IPDS-1 diffractometer
Absorption correction: numerical (*X-SHAPE* and *X-RED32*; Stoe & Cie, 2008) ▶ *T*_min_ = 0.165, *T*_max_ = 0.321
4502 measured reflections
657 independent reflections
638 reflections with *I* > 2σ(*I*)
*R*_int_ = 0.055

#### Refinement

*R*[*F* ^2^ > 2σ(*F* ^2^)] = 0.029
*wR*(*F* ^2^) = 0.073
*S* = 1.15
657 reflections
51 parameters
H-atom parameters constrained
Δρ_max_ = 0.40 e Å^−3^
Δρ_min_ = −0.81 e Å^−3^

### 

Data collection: *X-AREA* (Stoe & Cie, 2008) ▶; cell refinement: *X-AREA* ▶; data reduction: *X-AREA* ▶; program(s) used to solve structure: *SHELXS97* (Sheldrick, 2008 ▶); program(s) used to refine structure: *SHELXL97* (Sheldrick, 2008 ▶); molecular graphics: *XP* in *SHELXTL* (Sheldrick, 2008 ▶) and *DIAMOND* (Brandenburg, 2011 ▶); software used to prepare material for publication: *SHELXL97*.

## Supplementary Material

Crystal structure: contains datablock(s) I, global. DOI: 10.1107/S1600536811023129/wm2498sup1.cif

Structure factors: contains datablock(s) I. DOI: 10.1107/S1600536811023129/wm2498Isup2.hkl

Additional supplementary materials:  crystallographic information; 3D view; checkCIF report

## Figures and Tables

**Table:** 

Zn1—N1	2.033 (3)
Zn1—N11	2.287 (2)

**Table:** 

N1—C1—Se1	178.6 (3)

**Fig. 1.**
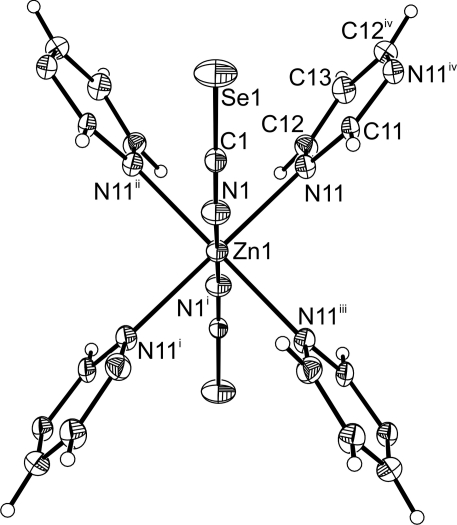
: Part of the crystal structure of the title compund, showing the coordination around Zn2+, with labelling and displacement ellipsoids drawn at the 50% probability level. [Symmetry codes: i = -x, -y + 1, -z + 1; ii = -x, y, z; iii = x, -y + 1, -z + 1; iv = -x + 1/2, y, -z + 3/2.]

**Fig. 2.**
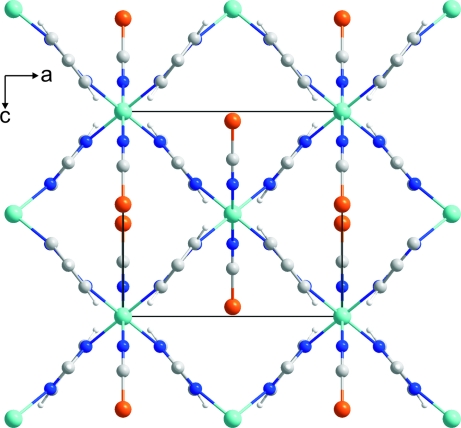
: Packing diagram of the title compound with view along the crystallographic b axis onto the polymeric layer (aqua = zinc; orange = selenium; blue = nitrogen; grey = carbon; light-grey = hydrogen).

## supplementary crystallographic information

### Comment

The structure determination of the title compound was performed as part of a project on the synthesis of new coordination polymers based on transition metal thiocyanates and the investigations of their thermal degradation products (Bhosekar *et al.*, 2010; Wriedt *et al.*, 2009; Wriedt & Näther, 2010)). Within this project we have reacted zinc(II) nitrate with potassium selenocyanate and pyrimidine in water, which leads to a single phase formation of the title compound, poly[bis(selenocyanato-κ*N*)-bis(µ_2_-pyrimidine-*N*,*N*')\ zinc].

The title compound is isotypic with its zinc, manganese(II), iron(II), cobalt(II) and nickel(II) thiocyanato coordination polymer analogues (Bhosekar *et al.*, 2010; Lloret *et al.*, 1998; Lloret *et al.*, 1999; Wriedt *et al.*, 2009; Wriedt & Näther, 2010). In the crystal structure the zinc atoms are surrounded by six N-atoms of four pyrimidine ligands and two N-bonded selenocyanato anions in mutually *trans* orientations in a slightly distorted octahedral geometry (Fig. 1). The pyrimidine ligands bridge the metal cations forming layers which extend along the *ac* plane (Fig. 2). These layers are stacked in the direction of the crystallographic *b* axis. The Zn—Zn intralayer separation amounts to 6.4352 (4) Å, whereas the shortest Zn—Zn interlayer separation is 9.4422 (5) Å.

### Experimental

The title compound was prepared by the reaction of 74.35 mg Zn(NO_3_)_2_ (0.25 mmol), 64.8 mg KSeCN (0.45 mmol) and 78.8 µ*L* pyrimidine (0.50 mmol) in 1.00 ml water at RT in a closed 3 ml snap cap vial. After one week colourless needles of the title compound were obtained.

### Refinement

All H atoms were located in difference map but were positioned with idealized geometry and were refined isotropically with *U*_eq_(H) = 1.2 *U*_eq_(C) of the parent atom using a riding model with C—H = 0.95 Å.

### Crystal data

**Table:**

[Zn(NCSe)_2_(C_4_H_4_N_2_)_2_]	*Z* = 4
*M**_r_* = 435.51	*F*(000) = 832
Orthorhombic, *C**m**c**a*	*D*_x_ = 2.094 Mg m^−^^3^
Hall symbol: -C 2bc 2	Mo *K*α radiation, λ = 0.71073 Å
*a* = 9.4025 (9) Å	µ = 7.04 mm^−^^1^
*b* = 16.7146 (10) Å	*T* = 200 K
*c* = 8.7886 (5) Å	Needle, colourless
*V* = 1381.21 (17) Å^3^	0.28 × 0.22 × 0.16 mm

### Data collection

**Table:**

Stoe IPDS-1 diffractometer	657 independent reflections
Radiation source: fine-focus sealed tube	638 reflections with *I* > 2σ(*I*)
graphite	*R*_int_ = 0.055
φ scans	θ_max_ = 25.5°, θ_min_ = 3.4°
Absorption correction: numerical (*X-SHAPE* and *X-RED32*; Stoe & Cie, 2008)	*h* = −11→11
*T*_min_ = 0.165, *T*_max_ = 0.321	*k* = −19→17
4502 measured reflections	*l* = −9→10

### Refinement

**Table:**

Refinement on *F*^2^	Primary atom site location: structure-invariant direct methods
Least-squares matrix: full	Secondary atom site location: difference Fourier map
*R*[*F*^2^ > 2σ(*F*^2^)] = 0.029	Hydrogen site location: inferred from neighbouring sites
*wR*(*F*^2^) = 0.073	H-atom parameters constrained
*S* = 1.15	*w* = 1/[σ^2^(*F*_o_^2^) + (0.0424*P*)^2^ + 2.801*P*] where *P* = (*F*_o_^2^ + 2*F*_c_^2^)/3
657 reflections	(Δ/σ)_max_ = 0.001
51 parameters	Δρ_max_ = 0.40 e Å^−^^3^
0 restraints	Δρ_min_ = −0.81 e Å^−^^3^

### Special details

**Table:**

Geometry. All e.s.d.'s (except the e.s.d. in the dihedral angle between two l.s. planes) are estimated using the full covariance matrix. The cell e.s.d.'s are taken into account individually in the estimation of e.s.d.'s in distances, angles and torsion angles; correlations between e.s.d.'s in cell parameters are only used when they are defined by crystal symmetry. An approximate (isotropic) treatment of cell e.s.d.'s is used for estimating e.s.d.'s involving l.s. planes.
Refinement. Refinement of *F*^2^ against ALL reflections. The weighted *R*-factor *wR* and goodness of fit *S* are based on *F*^2^, conventional *R*-factors *R* are based on *F*, with *F* set to zero for negative *F*^2^. The threshold expression of *F*^2^ > σ(*F*^2^) is used only for calculating *R*-factors(gt) *etc*. and is not relevant to the choice of reflections for refinement. *R*-factors based on *F*^2^ are statistically about twice as large as those based on *F*, and *R*- factors based on ALL data will be even larger.

### Fractional atomic coordinates and isotropic or equivalent isotropic displacement parameters (Å^2^)

**Table:**

	*x*	*y*	*z*	*U*_iso_*/*U*_eq_	
Zn1	0.0000	0.5000	0.5000	0.0172 (2)	
N1	0.0000	0.40527 (19)	0.6452 (4)	0.0220 (7)	
C1	0.0000	0.3787 (2)	0.7666 (4)	0.0164 (7)	
Se1	0.0000	0.34051 (3)	0.95337 (5)	0.0329 (2)	
N11	0.1681 (2)	0.56159 (13)	0.6471 (2)	0.0191 (5)	
C11	0.2500	0.5254 (2)	0.7500	0.0190 (7)	
H11	0.2500	0.4685	0.7500	0.023*	
C12	0.1740 (3)	0.64196 (17)	0.6445 (3)	0.0216 (6)	
H12	0.1240	0.6699	0.5671	0.026*	
C13	0.2500	0.6849 (2)	0.7500	0.0227 (8)	
H13	0.2500	0.7417	0.7500	0.027*	

### Atomic displacement parameters (Å^2^)

**Table:**

	*U*^11^	*U*^22^	*U*^33^	*U*^12^	*U*^13^	*U*^23^
Zn1	0.0157 (3)	0.0225 (4)	0.0133 (4)	0.000	0.000	0.0033 (2)
N1	0.0240 (16)	0.0238 (17)	0.0182 (16)	0.000	0.000	0.0017 (13)
C1	0.0123 (15)	0.0190 (17)	0.0178 (18)	0.000	0.000	−0.0030 (14)
Se1	0.0494 (4)	0.0337 (3)	0.0156 (3)	0.000	0.000	0.00690 (16)
N11	0.0147 (11)	0.0240 (12)	0.0186 (11)	−0.0001 (8)	−0.0012 (9)	0.0001 (8)
C11	0.0113 (15)	0.0247 (19)	0.0210 (18)	0.000	−0.0013 (14)	0.000
C12	0.0186 (13)	0.0262 (14)	0.0198 (13)	0.0001 (11)	−0.0012 (11)	0.0026 (10)
C13	0.0192 (19)	0.0212 (19)	0.028 (2)	0.000	−0.0011 (16)	0.000

### Geometric parameters (Å, °)

**Table:**

Zn1—N1^i^	2.033 (3)	N11—C11	1.333 (3)
Zn1—N1	2.033 (3)	N11—C12	1.345 (4)
Zn1—N11	2.287 (2)	C11—N11^iv^	1.333 (3)
Zn1—N11^i^	2.287 (2)	C11—H11	0.9500
Zn1—N11^ii^	2.287 (2)	C12—C13	1.373 (3)
Zn1—N11^iii^	2.287 (2)	C12—H12	0.9500
N1—C1	1.156 (5)	C13—C12^iv^	1.373 (3)
C1—Se1	1.761 (4)	C13—H13	0.9500
			
N1^i^—Zn1—N1	180.00 (11)	C1—N1—Zn1	151.5 (3)
N1^i^—Zn1—N11	90.23 (9)	N1—C1—Se1	178.6 (3)
N1—Zn1—N11	89.77 (9)	C11—N11—C12	116.2 (2)
N1^i^—Zn1—N11^i^	89.77 (9)	C11—N11—Zn1	125.3 (2)
N1—Zn1—N11^i^	90.23 (9)	C12—N11—Zn1	117.95 (17)
N11—Zn1—N11^i^	180.0	N11^iv^—C11—N11	126.0 (4)
N1^i^—Zn1—N11^ii^	90.23 (9)	N11^iv^—C11—H11	117.0
N1—Zn1—N11^ii^	89.77 (9)	N11—C11—H11	117.0
N11—Zn1—N11^ii^	87.46 (11)	N11—C12—C13	122.2 (3)
N11^i^—Zn1—N11^ii^	92.54 (11)	N11—C12—H12	118.9
N1^i^—Zn1—N11^iii^	89.77 (9)	C13—C12—H12	118.9
N1—Zn1—N11^iii^	90.23 (9)	C12—C13—C12^iv^	117.0 (4)
N11—Zn1—N11^iii^	92.54 (11)	C12—C13—H13	121.5
N11^i^—Zn1—N11^iii^	87.45 (11)	C12^iv^—C13—H13	121.5
N11^ii^—Zn1—N11^iii^	180.0		

Symmetry codes: (i) −*x*, −*y*+1, −*z*+1; (ii) −*x*, *y*, *z*; (iii) *x*, −*y*+1, −*z*+1; (iv) −*x*+1/2, *y*, −*z*+3/2.
